# Systems Pharmacology Uncovers the Multiple Mechanisms of Xijiao Dihuang Decoction for the Treatment of Viral Hemorrhagic Fever

**DOI:** 10.1155/2016/9025036

**Published:** 2016-04-27

**Authors:** Jianling Liu, Tianli Pei, Jiexin Mu, Chunli Zheng, Xuetong Chen, Chao Huang, Yingxue Fu, Zongsuo Liang, Yonghua Wang

**Affiliations:** ^1^College of Life Science, Northwest University, Xi'an, Shaanxi 710069, China; ^2^Center of Bioinformatics, College of Life Science, Northwest A&F University, Yangling, Shaanxi 712100, China; ^3^College of Life Science, Zhejiang Sci-Tech University, Hangzhou, Zhejiang 310000, China

## Abstract

*Background.* Viral hemorrhagic fevers (VHF) are a group of systemic diseases characterized by fever and bleeding, which have posed a formidable potential threat to public health with high morbidity and mortality. Traditional Chinese Medicine (TCM) formulas have been acknowledged with striking effects in treatment of hemorrhagic fever syndromes in China's history. Nevertheless, their accurate mechanisms of action are still confusing.* Objective.* To systematically dissect the mechanisms of action of Chinese medicinal formula Xijiao Dihuang (XJDH) decoction as an effective treatment for VHF.* Methods.* In this study, a systems pharmacology method integrating absorption, distribution, metabolism, and excretion (ADME) screening, drug targeting, network, and pathway analysis was developed.* Results.* 23 active compounds of XJDH were obtained and 118 VHF-related targets were identified to have interactions with them. Moreover, systematic analysis of drug-target network and the integrated VHF pathway indicate that XJDH probably acts through multiple mechanisms to benefit VHF patients, which can be classified as boosting immune system, restraining inflammatory responses, repairing the vascular system, and blocking virus spread.* Conclusions.* The integrated systems pharmacology method provides precise probe to illuminate the molecular mechanisms of XJDH for VHF, which will also facilitate the application of traditional medicine in modern medicine.

## 1. Introduction

Viral hemorrhagic fevers (VHF) are a group of systemic diseases caused by certain viruses, such as Ebola, Lassa, Dengue, and Crimean-Congo hemorrhagic fever viruses. Patients with VHF show the common cardinal symptoms, including fever, hemorrhages, and shock [1]. Data obtained over the past years indicate that these diseases are characterized by intense inflammatory responses with generalized signs of increased vascular permeability, severely impaired immune functions, diffuse vascular dysregulation, and coagulation abnormalities [2, 3]. VHF are generally prevalent in developing countries, which have posed a serious public health threat with high mortality, morbidity, and infectivity in recent years [4]. Currently, many large pharmaceutical companies are pursuing an effective antiviral therapy for VHF. Although the broad-spectra antiviral drug ribavirin is approved for treatment of several types of VHF, there remains a need for a safe and more effective medication to replace the antiviral drug [5].

Traditional Chinese Medicine (TCM) formulas consisting of complex mixtures of multiple plants play an outstanding role in the treatment of various acute infectious diseases because of the pharmacological and pharmacokinetic synergistic effects of the abundant bioactive ingredients [6]. A series of TCM prescriptions for hemorrhagic fever syndromes have been described in history [7, 8]. For example, XJDH is a famous TCM formula for treating hemorrhagic fever syndromes [9]. XJDH originally comes from “Prescriptions Worth A Thousand Gold” which is written by the “Medicinal King” Sun Simiao in the Tang Dynasty (around 700 AD) [10, 11]. The components of the formula include* Rhino horn* (substituted by* Buffalo Horn* now, Shui Niujiao in Chinese),* Rehmannia dried* rhizome (Sheng Dihuang in Chinese),* Paeonia lactiflora* Pall. (Shao Yao in Chinese), and* Paeonia suffruticosa* Andr. (Mu Danpi in Chinese). Actually, XJDH has been normally used for cooling the blood for hemostasis, stopping bleeding accompanied with fever, removing toxic substances, and treating the cases of high fever and sweating, spontaneous bleeding, hemoptysis, and nosebleeds [12, 13]. Although the therapeutic efficiency of XJDH in the treatment of VHF is attractive, several fundamental questions are still unclear. What are the potential active ingredients of XJDH? What are the underlying molecular mechanisms of action of the formula in the treatment of VHF? What are the precise targets of these medicines? Since the multiple components-multiple targets interaction model of TCM formulas, traditional experimental research methods show up the shortcomings of long-term investment.

Fortunately, as an emerging discipline, systems pharmacology provides a new way to solve the complex pharmacological problems [14]. Systems pharmacology integrates pharmacokinetic data (ADME/T characteristics of a drug) screening together with targets prediction, networks, and pathways analyses to explore the drug actions from molecular and cellular levels to tissue and organism levels. It also provides an analysis platform for decoding molecular mechanisms of TCM formulas. In our previous work, a series of systems pharmacology methods have been exploited to uncover the underlying mechanisms of action of TCM formulas for cardiovascular diseases, depression, and cancer [15–17].

**Figure 1:**
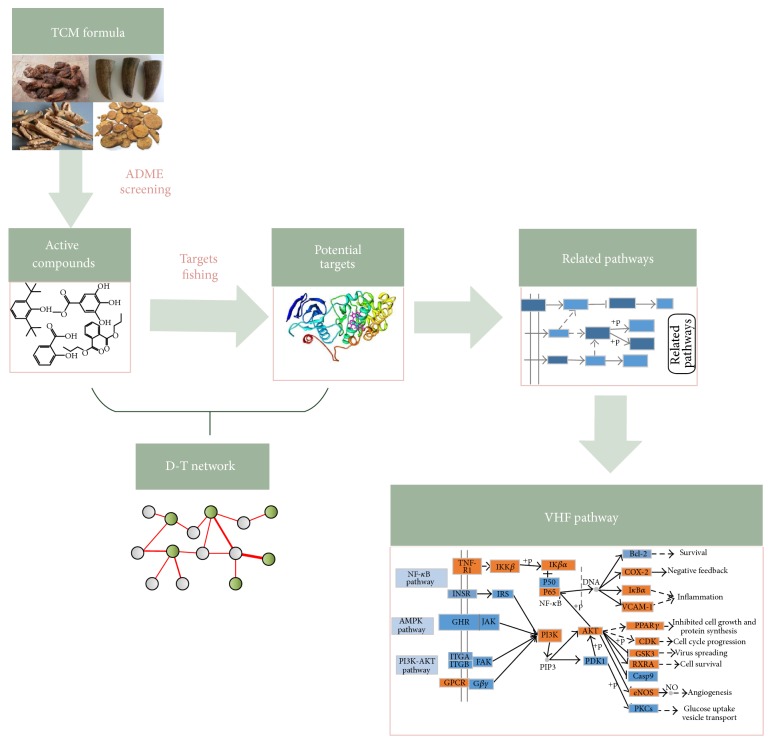
The detailed flowchart of the systems pharmacology method.

The purpose of the present study is to investigate the underlying molecular mechanisms of XJDH in treating VHF based on systems pharmacology method. Firstly, four pharmacokinetic models, including oral bioavailability (OB), drug-likeness (DL), Caco-2 permeability, and drug half-life (HL), were employed to filter out the potential active ingredients with favorable ADME profiles from XJDH. Then, based on an integrated target prediction method which combined the biological and mathematical models, the corresponding targets of these active ingredients were identified. Finally, the network pharmacology and VHF-related signaling pathways analysis was carried out to systematically disclose the underlying interactions between drugs, target proteins, and pathways. The detailed flowchart of the systems pharmacology method is shown in Figure 1.

## 2. Materials and Methods

### 2.1. Active Compounds Database

All chemicals of these four medicines in XJDH were manually collected from a wide-scale text mining and our in-house developed database: the Traditional Chinese Medicine Systems Pharmacology Database (TCMSP, http://lsp.nwsuaf.edu.cn/tcmsp.php) [18]. In order to obtain the potential active compounds from these medicines, we applied a method incorporating OB, DL, Caco-2 permeability, and drug HL evaluation in this work.

#### 2.1.1. OB Prediction

OB is defined as “the ratio of how many active components absorbed into the circulatory system to play a role at the site of action.” OB is one of the vital pharmacokinetic profiles in active compounds screening processes. In this work, the OB screening was calculated by a robust in-house system, OBioavail1.1 [19], and components with OB ≥ 30% were selected as the candidate molecules for further study. The following two basic sections describe the design principles of the threshold: (1) information from the studied medicines is obtained as much as possible using the least number of compounds and (2) the established model can be elucidated within reason by the reported pharmacological data [6].

#### 2.1.2. DL Prediction

DL generally means “molecule which holds functional groups and/or has physical properties consistent with the majority of known drugs” [20]. In this study, we performed a self-constructed model pre-DL (predicts drug-likeness) based on the molecular descriptors and Tanimoto coefficient [21]. The DL index of the compounds was calculated by Tanimoto coefficient defined as

(1) $$\begin{array}{c} T\left(\;A,B\;\right)=\frac{A\cdot B}{{\left\|A\right\|}^{2}+{\left\|B\right\|}^{2}-A\cdot B}, \end{array}$$

where *A* is the molecular descriptors of herbal compounds, *B* represents the average molecular properties of all compounds in DrugBank database (http://www.drugbank.ca/) [22]. The DL ≥ 0.18 (average value for DrugBank) was defined as the criterion to select those drug-like compounds which are chemically suitable for drugs.

#### 2.1.3. Caco-2 Permeability Prediction

The majority of orally administered drugs absorption occurs in the small intestine where the surface absorptivity greatly improves with the presence of villi and microvilli [23]. Previously, researchers have developed a quantity of* in silico* drug absorption models using* in vitro* Caco-2 permeability in drug discovery and development processes [24]. In this study, based on 100 drug molecules with satisfactory statistical results, a robust* in silico* Caco-2 permeability prediction model pre-Caco-2 (predicts Caco-2 permeability) was employed to predict the compound's intestinal absorption [25]. Finally, on the account of the fact that compounds with Caco-2 value less than 0 are not permeable, in the study, the threshold of Caco-2 permeability was set to 0.

#### 2.1.4. Drug HL Prediction

An* in silico* pre-HL (predicts half-life) has been developed in our previous work to calculate the drug HL by using the C-partial least square (C-PLS) algorithm which is supported by 169 drugs with known half-life values [26–28]. HL evaluates the time needed for compounds in the body to fall by half, and components with long HL were selected as the candidate molecules.

In order to obtain the potential active ingredients, the screening principle was defined as follows: OB ≥ 30%; Caco-2 ≥ 0; DL ≥ 0.18; or long HL.

### 2.2. Drug Targeting

Apart from screening out the active compounds, the therapeutic targets exploration is also a vital stage. Firstly, the potential targets exploration was fulfilled based on the systematic drug targeting tool (SysDT) as described in our previous work. Based on two mathematical tools, Random Forest (RF) and Support Vector Machine (SVM), the method can comprehensively ascertain the compound-target interaction profiles [29]. These two models exert great property of predicting the drug-target mutual effects with a concordance of 82.83%, a sensitivity of 81.33%, and a specificity of 93.62%. In this work, the compound-target interactions with SVM score ≥ 0.8 and the RF score ≥ 0.7 were selected for further research. Secondly, a recently developed computational model named weighted ensemble similarity (WES) was also introduced to detect drug direct targets [30]. For internal validation, this model performed remarkably well in predicting the binding (average sensitivity 72%, SEN) and the nonbinding (average specificity 82%, SPE) patterns, with the average areas under the receiver operating curves (ROC, AUC) of 85.2% and an average concordance of 77.5%. Thirdly, the obtained protein targets were mapped to the database UniProt (http://www.uniprot.org/) for normalization [31]. Finally, in order to identify and analyze the specific biological properties of the potential targets, the Gene Ontology (GO) biological processes were introduced to dissect target genes in a hierarchically structured way based on biological terms [32]. The GlueGO, a Cytoscape plug-in, was utilized to interpret the biology processes of large lists of genes.

### 2.3. Network Construction and Analysis

In order to explore the multiple mechanisms of action of XJDH for VHF, currently we analyzed the relationship between candidate compounds and potential targets by constructing the drug-target network (D-T network), in which all active compounds are connected to their targets. The network was generated by Cytoscape 2.8.1 [33]. In the network, compounds and targets are represented by nodes, while the interactions between them are represented by edges. In addition, a vital topological parameter, namely, degree was analyzed by the plugin NetworkAnalyzer of Cytoscape [34]. The degree of a node is defined as the number of edges connected to the node.

### 2.4. Pathway Construction and Analysis

At the pathway level, in order to probe into the action mechanisms of the formula for VHF, an incorporated “VHF pathway” was established based on the current knowledge of VHF pathology. Firstly, the obtained human target proteins were collected to be input into the Kyoto Encyclopedia of Genes and Genomes (KEGG, http://www.kegg.jp/) database to acquire the information of pathways. Then, based on the obtained information of basic pathways, we assembled an incorporated “VHF pathway” by picking out closely linked pathways related with VHF pathology.

## 3. Results

### 3.1. Active Compounds Screening

We employed four ADME parameters to screen out the potential active components of XJDH. As a result, from the 136 compounds of XJDH (as shown in supporting information, Table S1, in Supplementary Material available online at http://dx.doi.org/10.1155/2016/9025036), a total of 20 active compounds pass through the criteria of OB ≥ 30%, Caco-2 ≥ 0, DL ≥ 0.18, or long HL. Besides, in order to obtain a more accurate result, some certain rejected compounds, which have relatively poor pharmacokinetic properties, but are the most abundant and active ingredients of certain herbs, were also selected as the active components for further research. For example, although catalpol (MOL108) has poor OB, Caco-2, and HL properties, it has been reported to be rich in the roots of* Rehmannia dried* rhizome. And rehmaglutin D (MOL116) and paeoniflorin (MOL046) with poor OB (14.43%) take a large proportion in* Paeonia lactiflora* Pall. Thus, the three compounds were also retained for further analysis. Finally, a total of 23 active ingredients were obtained in this study (as shown in Table 1).

For all these 23 ingredients, many of them have been reported to demonstrate significant biologic activity including anti-inflammatory, antivirus, antipyretic, and immune-regulatory activities and protection effect of vascular endothelial cell. For instance, methyl gallate (MOL006, OB = 30.91%, Caco-2 = 0.26, and long HL), obtained from* Paeonia lactiflora* Pall., shows antivirus activity by interacting with virus proteins and altering the adsorption and penetration of the virion [35]. Salicylic acid (MOL045, OB = 32.13%, Caco-2 = 0.63, and long HL) and paeoniflorin (MOL046) with poor OB from* Paeonia lactiflora* Pall. exhibit antipyretic, anti-inflammatory, and immune-regulatory activities [36, 37]. In addition, kaempferol (MOL060, OB = 69.61%, Caco-2 = 0.15, DL = 0.24, and long HL), paeonol (MOL072, OB = 30.98%, Caco-2 = 0.91, long HL), and eugenol (MOL070, OB = 44.47%, Caco-2 = 1.36, and long HL), the main active compounds of the radix of* Paeonia suffruticosa* Andr., have been reported to have potential therapeutic effect for inflammation and vascular injury disorders [38–40]. Besides, it is worth noting that *β*-sitosterol (MOL018, OB = 36.9%, DL = 0.75) is a common ingredient of* Rehmannia dried* rhizome,* Paeonia lactiflora* Pall., and* Paeonia suffruticosa* Andr., indicating that these active compounds may show synergetic pharmacological effects on VHF.

### 3.2. Drug Targeting and Functional Analysis

Traditional information retrieval approaches of therapeutic targets of drugs are expatiatory and complicated [41]. To overcome this barrier, we introduced our previous developed target prediction model [29, 30] to dissect interactions between drugs and proteins. As a result, 23 candidate compounds are linked with 118 candidate targets (as shown in Table 2). The results show that many components simultaneously can act on more than one target and many targets can connect to all of the four medicines, demonstrating the promiscuous actions and analogous pharmacological effects of the bioactive molecules. For instance, kaempferol (MOL060) not only serves as the restrainer of Prostaglandin G/H synthase 2 [42] but also acts as the inhibitor of tumor necrosis factor [43]. And *β*-sitosterol (MOL018), which is shared by* Rehmannia dried* rhizome,* Paeonia lactiflora* Pall., and* Paeonia suffruticosa* Andr., acts as the activator of estrogen receptor [44] and transcription factor AP-1 [45]. Meanwhile, the results show that different drugs in XJDH can immediately impact on the common targets such as DNA ligase 1 (LIG1), indicating the synergism or cumulative effects of the drug molecules.

**Figure 2:**
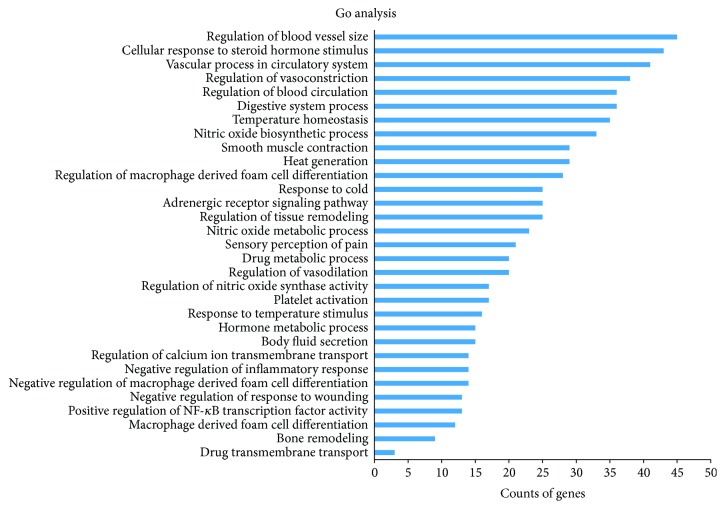
ClueGO analysis of the potential targets. *y*-axis shows significantly enriched “biological process” (BP) categories in GO relative to the target genes, and *x*-axis shows the counts of targets.

In general, vascular system, particularly the endothelium, plays a key role in VHF development [3]. A strong inflammatory response characterized by high circulating concentrations of cytokines and chemokines occurs early during the VHF infectious process [46]. And the patients' immune functions might also be severely impaired; innate defenses are further hindered by the loss of natural killer cells [47]. The relevant biological processes of above targets were revealed by GlueGO (as shown in supporting information, Table S2).  Figure 2 provides primary biological processes of these targets by cluster analysis. It is interesting to note that these targets are involved in a variety of biological processes including regulation of macrophage derived from cell differentiation, regulation of vasoconstriction and vasodilation, nitric oxide biosynthetic process, and vascular process in circulatory system. These biological processes largely fall into three groups: controlling inflammation response, modulating the immune system, and accommodation of vascular system. For example, peroxisome proliferator-activated receptor gamma (PPAR*γ*), tumor necrosis factor receptor superfamily member 1A (TNFRSF1A), beta-2 adrenergic receptor (ADRB2), and so forth are involved in the regulation of acute inflammatory response. Nitric oxide synthase, inducible (NOS2), dipeptidyl peptidase 4 (DPP4), and so forth are associated with regulation of immune effector process, while nitric oxide synthase, endothelial (NOS3), NOS2, Krüppel-like factor 5 (KLF5), and so forth are directly connected to blood vessel remodeling and blood vessel morphogenesis. These suggest that XJDH might exert the therapeutic effect on VHF mainly through anti-inflammation, enhancing immunity and vascular repair therapy.

### 3.3. Drug-Target Network Construction and Analysis

**Figure 3:**
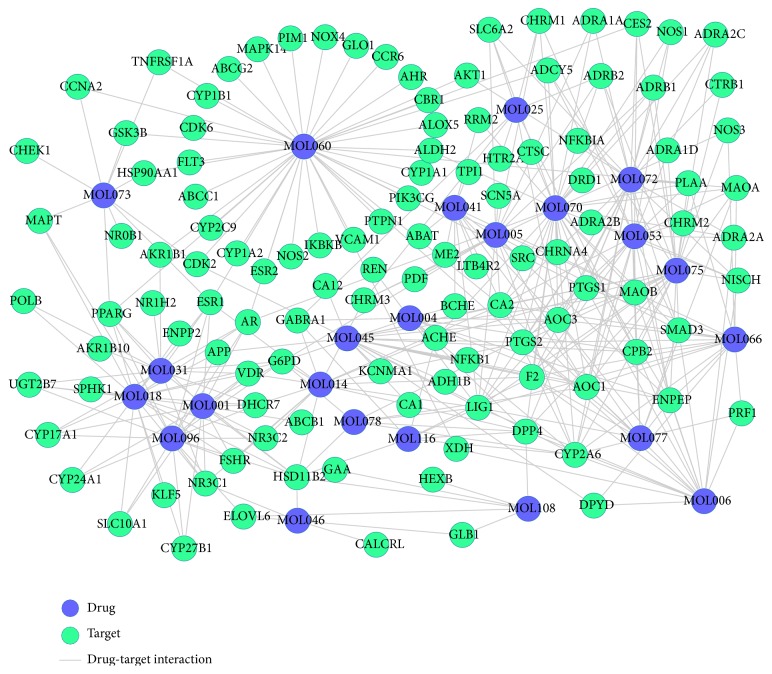
D-T network. The blue circles represent candidate compounds in XJDH, while the green circles represent target proteins, and each edge represents the interaction between them.

As shown in Figure 3, D-T network is constructed including 141 nodes (23 active compounds and 118 potential targets) and 382 edges. The degrees of the candidate compounds are shown in Table 1; this provides us with an intuitionistic concept to distinguish those highly connected vital compounds or targets from the others in the network. The results of network analysis show that 18 out of 23 candidate compounds are linked with more than ten targets, among which kaempferol (MOL060) displays the highest number of target interactions (degree = 41), followed by eugenol (MOL070, degree = 34) and paeonol (MOL072, degree = 27). This confirms the multitarget properties of herbal compounds. We speculate that the top three ingredients might be the crucial elements in the treatment of VHF. For instance, kaempferol (MOL060) is predicted to interact with 41 targets like calcitonin gene-related peptide type 1 receptor (MAPK14), PPAR*γ*, and phosphatidylinositol-4,5-bisphosphate 3-kinase catalytic subunit gamma isoform (PIK3CG). MAPK14 takes part in the vascular endothelial growth factor (VEGF) synthesis through the mediation of angiotensin II [48]. VEGF can induce angiogenesis and improve the increased vascular permeability, so as to prevent bleeding in patients with VHF [49]. Besides, kaempferol is also found to significantly upregulate the transcriptional activity of PPAR*γ*, which acts as an inhibitor of inflammatory gene expression and vandalizes proinflammatory transcription factor signaling pathways in vascular cells [50]. Additionally, previous finding suggests that PIK3CG interacting with kaempferol can participate in inflammation processes and influence the innate immune system [51]. Thus, these key active ingredients of XJDH work mainly by modulating inflammatory factor, innate immune system, and VEGF.

Meanwhile, the results also show that one target can be hit by multiple compounds from different medicines, indicating synergism or summation effects of the formula. According to the D-T network analysis, 64 out of the 118 targets have at least two links with the components of different herbs. XJDH exerts its therapeutic effect for VHF by binding and regulating particular protein targets. For instance, prostaglandin G/H synthase 2 (PTGS2) is simultaneously targeted by 10 active compounds including eugenol (MOL070, from* Paeonia suffruticosa* Andr.), paeonol (MOL072, from* Paeonia suffruticosa* Andr.), and salicylic acid (MOL045, from* Paeonia lactiflora* Pall.). PTGS2 is possibly an effective marker of platelet dysfunction; a reduced PTGS2 expression in the VHF primate model cells could directly result in platelet dysfunction [52]. Fortunately, in agreement with our study, previous findings suggest that eugenol and paeonol can control the expression of PTGS2 through the suppression of NF-*κ*B in macrophage [53–55], so as to recover the function of thrombocyte. Study shows that severe disseminated intravascular coagulation is the mechanisms of bleeding in all VHF [3]. Prothrombin (F2), the precursor substances of clotting enzyme, plays a crucial role in optimizing the procoagulant activity through controlling the anticoagulant function of meizothrombin [56]. Thus, these 10 ingredients such as methyl gallate (MOL006, from* Paeonia lactiflora* Pall.), salicylic acid (MOL045, from* Paeonia lactiflora* Pall.), and apocynin (MOL053,* Paeonia suffruticosa* Andr.) interacting with F2 may be the key factors in the treatment of bleeding in patients with VHF. By analyzing the above D-T network, we can conclude that XJDH produces the healing efficacy for VHF probably by three different ways, intervening in the process of inflammation, boosting immune reaction, and repairing vascular system.

### 3.4. Pathway Analysis

**Figure 4:**
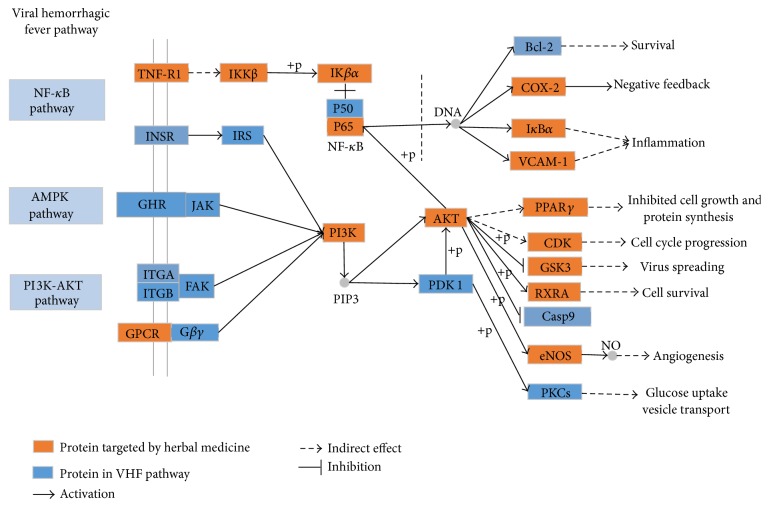
The VHF pathway and therapeutic modules.

To explore the integral regulation of XJDH for the treatment of VHF, we assembled an integrated “VHF pathway” (Figure 4) on the basis of the current knowledge of VHF pathogenesis. By means of inputting the obtained human target proteins into KEGG pathway database, result shows that 110 of the 118 targets can be mapped to the KEGG pathways, including NF-*κ*B signaling pathway, AMPK pathway, and PI3K-AKT signaling pathway. Now, three detailed therapeutic modules are provided (inflammation module, angiogenesis module, and virus spreading module).

#### 3.4.1. Inflammation Module

As shown in Figure 4, 7 key proteins targeted by XJDH are mapped onto a key inflammation process, namely, NF-*κ*B signaling pathway, indicating the anti-inflammatory action may play a vital role in the treatment of VHF. Patients with VHF have a strong inflammatory response with high inflammatory cytokines and chemokines levels such as IL-1*β*, TNF, and IL-6 in the early phase of VHF [46]. The expression of inflammatory cytokines (TNF-*α*, IL-1*β*, IL-6, and IL-8) is mediated by NF-*κ*B [57], and NF-*κ*B is one of the most important regulators of proinflammatory gene expression. The result demonstrates that paeoniflorin (MOL046) from* Paeonia lactiflora* Pall. and* Paeonia suffruticosa* Andr. can regulate transcription factor NF-*κ*B activity. Meanwhile, other researchers have verified that paeoniflorin can restrain the activation of the NF-*κ*B pathway via inhibiting I*κ*B kinase [58]. In addition, vascular adhesion molecule 1 (VCAM-1), a cell adhesion molecule, also plays an important role in the pathogenesis of inflammatory and immune processes [59]. Our work indicates that kaempferol (MOL060) and *β*-sitosterol (MOL018) can regulate the expression of inflammatory cytokines by targeting vascular adhesion molecule 1 (VCAM-1). Consequently, the foregoing analysis shows that XJDH has the effect of ameliorating the symptoms of inflammation disorders of patients with VHF.

#### 3.4.2. Virus Spreading Module

In Figure 4, the phosphoinositide-3 kinase (PI3K) pathway is a significant cell signaling pathway that regulates diverse cellular activities including cell proliferation, differentiation, apoptosis, and vesicular trafficking. Notably, our research shows that, in line with previous studies, kaempferol (MOL060) is predicted to modulate PI3Ks activity, and AKT is also a target for kaempferol (MOL060) and paeonol (MOL072) [60–62]. Besides, PI3Ks, a family of lipid kinases, can prevent hemorrhagic fever virus entry into host cells by regulating cellular activities of vesicular trafficking [63]. AKT is a major downstream effector of the PI3K pathway, and this target protein can control the expression of many molecules directly or indirectly. Moreover, evidence also suggests that activity of PI3K/Akt pathway is required for hemorrhagic fever virus intruding into the host cells [63]. Therefore, depressors of PI3K and AKT dramatically reduced the risk of hemorrhagic fever virus infection at an early step during the replication cycle. These above analyses show that XJDH could make effective control of hemorrhagic fever virus entry into cells, thus blocking virus spread by interfering with the PI3K-AKT signaling pathway.

#### 3.4.3. Angiogenesis Module

VHF is a severe multisystem syndrome characterized by diffuse vascular damage. The vascular system, particularly the vascular endothelium, seems to be directly and indirectly targeted by hemorrhagic fever viruses [3]. In the VHF pathway shown in Figure 4, PI3K pathway and AMPK pathway are involved in regulating the angiogenesis progress. We find out that apocynin (MOL053), kaempferol (MOL060), methyl salicylate (MOL066), and eugenol (MOL070) can affect the activity of endothelial NO synthase (eNOS) and then bring about NO production changes in endothelial cell. Moreover, at present, a large number of researches indicate that the synthesis of bioactive endothelium-derived NO is required for the progress of angiogenesis [64–66]. Therefore, the evidence presented enables us to reasonably conclude that the XJDH takes part in regulation of angiogenesis progress through PI3K signaling pathway and AMPK pathway.

## 4. Discussion

Actually, XJDH has been normally used for cooling the blood for hemostasis, stopping bleeding accompanied with fever, removing toxic substances, and treating the cases of high fever and sweating, spontaneous bleeding, hemoptysis, and nosebleeds [13, 67]. Although XJDH has been used historically for treating hemorrhagic fever syndromes, the specific bioactive molecules responsible for VHF and their precise mechanisms of action are still unclear. Thus, in this work, a systems pharmacology method combining the screening active components, drug targeting, network, and pathway analysis was carried out, so as to uncover the active ingredients, targets, and pathways of XJDH and systematically decipher its therapeutic mechanism of actions.

Our results show that 23 active ingredients were obtained from XJDH, and 118 potential targets were predicted. These manifest that the characteristics of XJDH are multicomponent botanical therapeutics and multitargets synergetic therapeutic effects. The GO analysis of targets and integrated D-T network analysis demonstrate the synergistic effects of XJDH for the treatment of VHF mainly through boosting of immune system, inhibiting inflammatory response, and repairing vascular system. Meanwhile, the integrated “VHF pathway” analysis in our work shows that XJDH might simultaneously regulate multitargets/pathways coupled with a range of therapeutic modules, for example, anti-inflammation, antivirus, and angiogenesis.

Now most researchers believe that VHF can be attributed to the simultaneous occurrence of multiple pathogenic mechanisms. They are mainly as follows: hemorrhagic fever virus infection stimulates macrophages to release cytokines, chemokines, and other mediators, causing fever, malaise, alterations in vascular function, and a shift in the coagulation system toward a procoagulant state, and immune functions might also be severely impaired [2]. Besides, hemorrhagic fever virus can target the vascular system directly and indirectly and cause endothelial activation and dysfunction [3]. In this study, we show here for the first time using GO enrichment analysis, network analysis, and integrated pathway analysis that XJDH significantly enriches target genes involved in reducing the inflammation response, enhancing immunity, combating the spreading virus, and preventing vascular dysfunction. And more experiments are needed to verify the validity of the results in further research works.

## 5. Conclusions

The result of this study provides bioactive ingredients, vital targets, and pathways of XJDH. We have come to the conclusion that the action mechanisms of XJDH for VHF mainly include restoring the immune system and enhancing immune response, ameliorating the symptoms of inflammation disorders, improving their vascular endothelial dysfunction, and combating the spreading virus. The systems pharmacology method established in our work provides preliminary clues that the multilayer networks of drug-target paradigm may be valuable for the modernization of TCM formulas at molecular level and then push forward their acceptance into mainstream medicine.

## Supplementary Material

Table S1: the 136 compounds information of XJDH.
Table S2: the relevant biological processes of the candidate targets.

## Figures and Tables

**Table 1:** 23 potential compounds of XJDH and their network parameters.

MOL_ID	Compounds	Structure	OB	Caco-2	DL	HL	Degree	Medicines
MOL001	Cholesterol	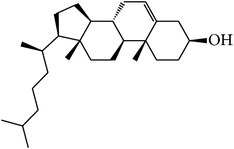	37.87	1.31	0.68	Long	16	*Buffalo Horn*

MOL004	2,2-Dimethylcyclohexanol	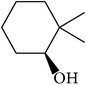	82.54	1.22	0.02	Long	12	*Paeonia lactiflora* Pall.

MOL005	Dibutylphenol	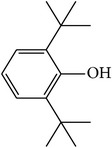	38.90	1.73	0.06	Long	15	*Paeonia lactiflora* Pall.

MOL006	Methyl gallate	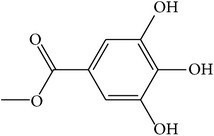	30.91	0.26	0.05	Long	17	*Paeonia lactiflora* Pall.

MOL014	(−)-Alpha-cedrene	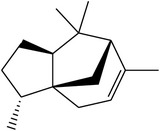	55.56	1.81	0.10	Long	16	*Paeonia lactiflora* Pall.

MOL018	*β*-Sitosterol	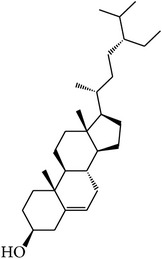	36.91	1.34	0.75	Short	21	*Paeonia lactiflora* Pall.,* Paeonia suffruticosa* Andr.,* Rehmannia dried* rhizome

MOL025	Dipropyl Phthalate	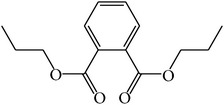	66.30	0.78	0.10	Long	8	*Paeonia lactiflora* Pall.

MOL031	Mairin	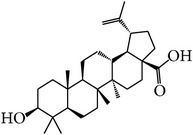	55.38	0.73	0.78	Short	14	*Paeonia lactiflora* Pall.

MOL041	Acetyl oxide	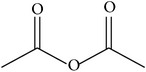	45.13	0.65	0	Long	12	*Paeonia lactiflora* Pall.

MOL045	Salicylic acid	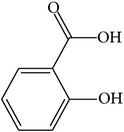	32.13	0.63	0.027	Long	19	*Paeonia lactiflora* Pall.

MOL046	Paeoniflorin	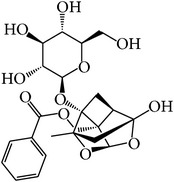	14.43	−1.38	0.79	Short	5	*Paeonia lactiflora* Pall.

MOL053	Apocynin	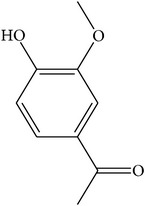	31.71	0.74	0.04	Long	22	*Paeonia suffruticosa* Andr.

MOL060	Kaempferol	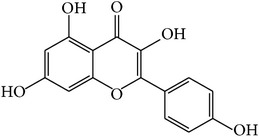	69.61	0.15	0.24	Long	41	*Paeonia suffruticosa* Andr.

MOL066	Methyl salicylate	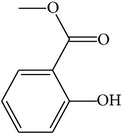	42.55	1.05	0.03	Long	17	*Paeonia suffruticosa* Andr.

MOL070	Eugenol	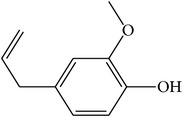	44.47	1.36	0.04	Long	34	*Paeonia suffruticosa* Andr.

MOL072	Paeonol	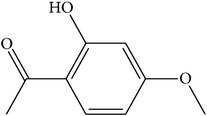	30.98	0.91	0.04	Long	27	*Paeonia suffruticosa* Andr.

MOL073	5-[[5-(4-Methoxyphenyl)-2-furyl]methylene]barbituric acid	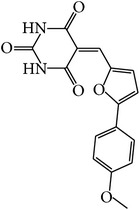	43.44	0.09	0.30	Long	10	*Paeonia suffruticosa* Andr.

MOL075	1-(2,3-Dihydroxy-4-methoxyphenyl)ethanone	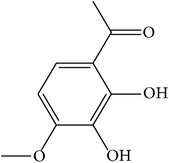	32.96	0.81	0.05	Long	25	*Paeonia suffruticosa* Andr.

MOL077	Vanillic acid	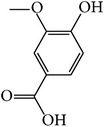	35.47	0.43	0.04	Long	13	*Paeonia suffruticosa* Andr.

MOL078	(1R)-(+)-Nopinone	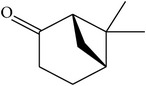	57.86	1.23	0.05	Long	7	*Paeonia suffruticosa* Andr.

MOL096	Stigmasterol	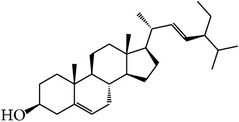	43.83	1.44	0.76	Short	18	*Rehmannia dried* rhizome

MOL108	Catalpol	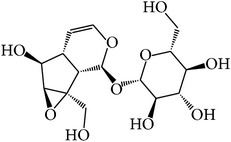	14.78	−2.10	0.44	Short	6	*Rehmannia dried* rhizome

MOL116	Rehmaglutin D	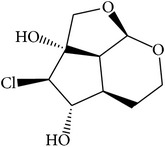	62.9	−0.31	0.1	Long	7	*Rehmannia dried* rhizome

**Table 2:** The VHF-related targets information.

UniProt ID	Name	Gene name	Species
O14920	Inhibitor of nuclear factor kappa-B kinase subunit beta	IKBKB	*Homo sapiens *
P19320	Vascular cell adhesion protein 1	VCAM1	*Homo sapiens *
P31749	RAC-alpha serine/threonine-protein kinase	AKT1	*Homo sapiens *
P19838	Nuclear factor NF-kappa-B p105 subunit	NFKB1	*Homo sapiens *
P25963	NF-kappa-B inhibitor alpha	NFKBIA	*Homo sapiens *
A1L156	LTB4R2 protein	LTB4R2	*Homo sapiens *
F1D8P7	Liver X nuclear receptor beta	NR1H2	*Homo sapiens *
O00748	Cocaine esterase	CES2	*Homo sapiens *
O14757	Serine/threonine-protein kinase Chk1	CHEK1	*Homo sapiens *
O15528	25-Hydroxyvitamin D-1 alpha hydroxylase, mitochondrial	CYP27B1	*Homo sapiens *
O43570	Carbonic anhydrase 12	CA12	*Homo sapiens *
O60218	Aldo-keto reductase family 1 member B10	AKR1B10	*Homo sapiens *
O95622	Adenylate cyclase type 5	ADCY5	*Homo sapiens *
P00325	Alcohol dehydrogenase 1B	ADH1B	*Homo sapiens *
P00734	Prothrombin	F2	*Homo sapiens *
P00797	Renin	REN	*Homo sapiens *
P00915	Carbonic anhydrase 1	CA1	*Homo sapiens *
P00918	Carbonic anhydrase 2	CA2	*Homo sapiens *
P03372	Estrogen receptor	ESR1	*Homo sapiens *
P04150	Glucocorticoid receptor	NR3C1	*Homo sapiens *
P04798	Cytochrome P450 1A1	CYP1A1	*Homo sapiens *
P05067	Amyloid beta A4 protein	APP	*Homo sapiens *
P05091	Aldehyde dehydrogenase, mitochondrial	ALDH2	*Homo sapiens *
P05093	Steroid 17-alpha-hydroxylase/17,20 lyase	CYP17A1	*Homo sapiens *
P05177	Cytochrome P450 1A2	CYP1A2	*Homo sapiens *
P06276	Cholinesterase	BCHE	*Homo sapiens *
P06746	DNA polymerase beta	POLB	*Homo sapiens *
P07550	Beta-2 adrenergic receptor	ADRB2	*Homo sapiens *
P07686	Beta-hexosaminidase subunit beta	HEXB	*Homo sapiens *
P07900	Heat shock protein HSP 90-alpha	HSP90AA1	*Homo sapiens *
P08172	Muscarinic acetylcholine receptor M2	CHRM2	*Homo sapiens *
P08183	Multidrug resistance protein 1	ABCB1	*Homo sapiens *
P08235	Mineralocorticoid receptor	NR3C2	*Homo sapiens *
P08588	Beta-1 adrenergic receptor	ADRB1	*Homo sapiens *
P08913	Alpha-2A adrenergic receptor	ADRA2A	*Homo sapiens *
P09917	Arachidonate 5-lipoxygenase	ALOX5	*Homo sapiens *
P10253	Lysosomal alpha-glucosidase	GAA	*Homo sapiens *
P10275	Androgen receptor	AR	*Homo sapiens *
P10636	Microtubule-associated protein tau	MAPT	*Homo sapiens *
P11229	Muscarinic acetylcholine receptor M1	CHRM1	*Homo sapiens *
P11309	Serine/threonine-protein kinase pim-1	PIM1	*Homo sapiens *
P11413	Glucose-6-phosphate 1-dehydrogenase	G6PD	*Homo sapiens *
P11413	Glucose-6-phosphate 1-dehydrogenase	G6PD	*Homo sapiens *
P11473	Vitamin D3 receptor	VDR	*Homo sapiens *
P11509	Cytochrome P450 2A6	CYP2A6	*Homo sapiens *
P11712	Cytochrome P450 2C9	CYP2C9	*Homo sapiens *
P12931	Proto-oncogene tyrosine-protein kinase Src	SRC	*Homo sapiens *
P14222	Perforin-1	PRF1	*Homo sapiens *
P14867	Gamma-aminobutyric-acid receptor subunit alpha-1	GABRA1	*Homo sapiens *
P15121	Aldose reductase	AKR1B1	*Homo sapiens *
P16152	Carbonyl reductase [NADPH] 1	CBR1	*Homo sapiens*
P16278	Beta-galactosidase	GLB1	*Homo sapiens*
P16662	UDP-glucuronosyltransferase 2B7	UGT2B7	*Homo sapiens*
P17538	Chymotrypsinogen B	CTRB1	*Homo sapiens*
P18031	Tyrosine-protein phosphatase nonreceptor type 1	PTPN1	*Homo sapiens*
P18089	Alpha-2B adrenergic receptor	ADRA2B	*Homo sapiens*
P18825	Alpha-2C adrenergic receptor	ADRA2C	*Homo sapiens*
P18858	DNA ligase 1	LIG1	*Homo sapiens*
P19438	Tumor necrosis factor receptor superfamily member 1A	TNFRSF1A	*Homo sapiens*
P19801	Amiloride-sensitive amine oxidase [copper-containing]	AOC1	*Homo sapiens*
P20248	Cyclin-A2	CCNA2	*Homo sapiens*
P20309	Muscarinic acetylcholine receptor M3	CHRM3	*Homo sapiens*
P21397	Amine oxidase [flavin-containing] A	MAOA	*Homo sapiens*
P21728	D(1A) dopamine receptor	DRD1	*Homo sapiens*
P22303	Acetylcholinesterase	ACHE	*Homo sapiens*
P23219	Prostaglandin G/H synthase 1	PTGS1	*Homo sapiens*
P23368	NAD-dependent malic enzyme, mitochondrial	ME2	*Homo sapiens*
P23945	Follicle-stimulating hormone receptor	FSHR	*Homo sapiens*
P23975	Sodium-dependent noradrenaline transporter	SLC6A2	*Homo sapiens*
P24941	Cell division protein kinase 2	CDK2	*Homo sapiens*
P25100	Alpha-1D adrenergic receptor	ADRA1D	*Homo sapiens*
P27338	Amine oxidase [flavin-containing] B	MAOB	*Homo sapiens*
P27487	Dipeptidyl peptidase 4	DPP4	*Homo sapiens*
P28223	5-Hydroxytryptamine 2A receptor	HTR2A	*Homo sapiens*
P29474	Nitric oxide synthase, endothelial	NOS3	*Homo sapiens*
P29475	Nitric oxide synthase, brain	NOS1	*Homo sapiens*
P31350	Ribonucleoside-diphosphate reductase subunit M2	RRM2	*Homo sapiens*
P33527	Multidrug resistance-associated protein 1	ABCC1	*Homo sapiens*
P35228	Nitric oxide synthase, inducible	NOS2	*Homo sapiens*
P35348	Alpha-1A adrenergic receptor	ADRA1A	*Homo sapiens*
P35354	Prostaglandin G/H synthase 2	PTGS2	*Homo sapiens*
P35869	Aryl hydrocarbon receptor	AHR	*Homo sapiens*
P36888	Receptor-type tyrosine-protein kinase FLT3	FLT3	*Homo sapiens*
P37231	Peroxisome proliferator-activated receptor gamma	PPARG	*Homo sapiens*
P43681	Neuronal acetylcholine receptor subunit alpha-4	CHRNA4	*Homo sapiens*
P47989	Xanthine dehydrogenase/oxidase [includes xanthine dehydrogenase]	XDH	*Homo sapiens*
P48736	Phosphatidylinositol-4,5-bisphosphate 3-kinase catalytic subunit gamma isoform	PIK3CG	*Homo sapiens*
P49841	Glycogen synthase kinase-3 beta	GSK3B	*Homo sapiens*
P51684	C-C chemokine receptor type 6	CCR6	*Homo sapiens*
P51843	Nuclear receptor subfamily 0 group B member 1	NR0B1	*Homo sapiens*
P53634	Dipeptidyl peptidase 1	CTSC	*Homo sapiens*
P60174	Triose-phosphate isomerase	TPI1	*Homo sapiens*
P80365	Corticosteroid 11-beta-dehydrogenase isozyme 2	HSD11B2	*Homo sapiens*
P80404	4-Aminobutyrate aminotransferase, mitochondrial	ABAT	*Homo sapiens*
P84022	Mothers against decapentaplegic homolog 3	SMAD3	*Homo sapiens*
Q00534	Cyclin-dependent kinase 6	CDK6	*Homo sapiens*
Q04760	Lactoylglutathione lyase	GLO1	*Homo sapiens*
Q07075	Glutamyl aminopeptidase	ENPEP	*Homo sapiens*
Q07973	1,25-Dihydroxyvitamin D(3) 24-hydroxylase, mitochondrial	CYP24A1	*Homo sapiens*
Q12791	Calcium-activated potassium channel subunit alpha-1	KCNMA1	*Homo sapiens*
Q12882	Dihydropyrimidine dehydrogenase [NADP(+)]	DPYD	*Homo sapiens*
Q13822	Ectonucleotide pyrophosphatase/phosphodiesterase family member 2	ENPP2	*Homo sapiens*
Q13887	Krüppel-like factor 5	KLF5	*Homo sapiens*
Q14524	Sodium channel protein type 5 subunit alpha	SCN5A	*Homo sapiens*
Q14973	Sodium/bile acid cotransporter	SLC10A1	*Homo sapiens*
Q16539	Mitogen-activated protein kinase 14	MAPK14	*Homo sapiens*
Q16602	Calcitonin gene-related peptide type 1 receptor	CALCRL	*Homo sapiens*
Q16678	Cytochrome P450 1B1	CYP1B1	*Homo sapiens*
Q16853	Membrane primary amine oxidase	AOC3	*Homo sapiens*
Q92731	Estrogen receptor beta	ESR2	*Homo sapiens*
Q96IY4	Carboxypeptidase B2	CPB2	*Homo sapiens*
Q9H5J4	Elongation of very long chain fatty acids protein 6	ELOVL6	*Homo sapiens*
Q9HBH1	Peptide deformylase, mitochondrial	PDF	*Homo sapiens*
Q9NPH5	NADPH oxidase 4	NOX4	*Homo sapiens*
Q9NYA1	Sphingosine kinase 1	SPHK1	*Homo sapiens*
Q9UBM7	7-Dehydrocholesterol reductase	DHCR7	*Homo sapiens*
Q9UNQ0	ATP-binding cassette subfamily G member 2	ABCG2	*Homo sapiens*
Q9Y263	Phospholipase A-2-activating protein	PLAA	*Homo sapiens*
Q9Y2I1	Nischarin	NISCH	*Homo sapiens*
